# Quality of Life of People with Cardiovascular Disease: A Descriptive Study

**DOI:** 10.31372/20190402.1045

**Published:** 2019

**Authors:** Renata Komalasari, Nurjanah, Maria M. Yoche

**Affiliations:** a Lecturer of Sekolah Tinggi Ilmu Kesehatan Tarumanagara, Jakarta, Indonesia; b Staff at Siloam Hospitals, Jakarta, Indonesia; c Lecturer of Faculty of Nursing, University of Pelita Harapan, Jakarta, Indonesia

**Keywords:** quality of life, aging, older people, cardiovascular disease, WHOQOL-BREF

## Abstract

In Indonesia, cardiovascular disease (CVD) ranks ninth among 22 types of diseases that result in death. Patients with history of CVD may experience various physical and emotional symptoms such as fatigue, edema, and sleeping difficulties that limit their physical and social activities which will in turn result in poor quality of life. Hospitalization and mortality has been associated with poor quality of life therefore people with history of CVD should be assessed appropriately to determine its impact on patients’ daily lives. This study aimed to examine the quality of life in older people with history of heart diseases. This study was conducted in an outpatient department of a private hospital in Tangerang, Banten Province, Indonesia. This quantitative, descriptive study involved 397 older patients. Data were collected via purposive sampling. Older patients with a history of CVD (cardiovascular diseases), aged 60–74 years, who could communicate and understand the Indonesian language and were not in a state that hindered completing a questionnaire were included in the study. Quality of life was measured using the WHOQOL-BREF questionnaire, which comprises four domains: physical health, psychological aspects, social relationships, and environment. Data were analyzed using a descriptive analysis. The results showed that 94% of respondents with a history of CVD had a good quality of life, with 85% having an adequate environmental aspect, 60.7% having active social relationships, 54.7% having good physical health, and 44.8% having a stable psychological condition. Despite having a history of CVD, most respondents in this study reported a good quality of life. However, the measurement tool used in this study measured quality of life in general. Future research should consider using a tool that is specific for measuring the quality of life of people with cardiac diseases.

## Introduction

An older person is defined as someone who is 60 years old and above. According to World Population Prospect (2017), globally, the number of people age 60 years and over will more than double, rising from 962 million in 2017 to 2.1 billion by 2050. Like countries across the world, Indonesia is facing an ageing structured population as it has more than 7 percent of older population (Kementerian Kesehatan Indonesia, 2017). There was an estimated 23.66 million older people in Indonesia in 2017, and this number is expected to be 27.08 million by 2020 and 48.19 million by 2035 (Kementerian Kesehatan Indonesia, 2017). Banten is amongst the 34 provinces in Indonesia with increased older population from 488,202 in 2010 to 599,090 in 2014 (Badan Pusat Statistik, 2016).

Advanced technology, decreased birth rates are among the contributing factors to increased life expectancy. Life expectancy in Indonesia has increased from 59.4 in 2000 to 62.1 in 2015 (World Health Organization, 2017), indicating good improvement of the overall health of the population. One of the diseases of older age is cardiovascular disease. Cardiovascular disease (CVD) refers to conditions affecting the heart or blood vessels. According to data from the World Health Organization, every year CVD is responsible for the death of 17.9 million people worldwide, which is equivalent to 31% of all deaths. According to the Centers for Disease Control and Prevention, in the United States, 1 in every 4 people die of heart disease every year which accounts for 610,000 people. In Indonesia, CVD causes 37% of all deaths, causing 18,000 disability-adjusted life years (DALYS) in 2012 and many years lost due to premature mortality and healthy life lost to disability (The George Institute for Global Health, 2017). Further, according to a medical record of inpatient rooms in a private hospital in Jakarta, 49,110 patients with a history of heart diseases came to the outpatient department of this hospital where this study was conducted.

Healthy life lost to disability due to CVD may make people with CVD experience poor quality of life (Rector et al., 2006). Various physical and emotional symptoms, such as dyspnea, fatigue, edema, difficulty sleeping, depression, and chest pain affiliated with CVD (Zambroski et al., 2005) may limit activities of daily life. Poor quality of life is related to high hospitalization and mortality rates (Konstam et al., 1996). Therefore, QOL of people with CVD should be assessed appropriately to determine its impact on their daily lives.

The World Health Organization Quality of Life (WHOQOL Group, 1996) defined quality of life as “individuals’ perceptions of their position in life in the context of the culture and value systems in which they live and in relation to their goals, expectations, standards and concerns” (World Health Organization Quality of Life (WHOQOL), 1996). Quality of life can be determined from the condition of the physical, psychological, social relationships, and environmental aspects (WHOQOL Group, 1997). The objective of this study was to examine the quality of life of patients with history of CVD in four domains (physical health, psychological health, social relationships, and environment). The results of this study will provide baseline data on the quality of life in patients with history of CVD.

## Methods

This study was conducted in an outpatient department (OPD) of a private hospital in West Jakarta, Indonesia, from January to August 2016. Ethics approval for this study was received from the Mochtar Riady Institute of Nanotechnology No. 127/MRIN-EC/VI/2016 as well as from the hospital management. Three months before the study was conducted, 1227 patients with a history of heart diseases were recorded present at the outpatient department in the hospital where the study was conducted. Therefore, in order to achieve power calculation of the statistical measurement, 397 older participants with a history of heart diseases were involved in the study. The investigator checked the medical records of the patients that came to the outpatient departments. Patients who had a history of essential hypertension, coronary heart disease, heart failure, and ischemic heart failure were then invited to be involved in the study.

This study used purposive sampling to select the study sample. The investigator was a nurse working in an outpatient department (OPD) in the hospital so she had the flexibility to move from one OPD to another in the hospital in order to select patients matching the study criteria. The OPDs visited by the investigator of the study consisted of the cardiovascular unit, internal medicine unit, neurology unit, and ophthalmology unit. The investigator then reviewed the participants’ medical records for a history of cardiovascular disease before inviting a patient to participate in the study. The investigator read each item in the questionnaire to each participant and recorded their responses in the questionnaire. Each participant took about 30 min to complete the questionnaire. The criteria for inclusion were age 60 to 74, having the ability to communicate and understand the Indonesian language, and not being in a state that may hinder participation as participants were required to complete a questionnaire, having consented to participate. Participants aged 60 to 74 years old were particularly sought because of limited funding of this study. The data were analyzed using a descriptive analysis.

The WHOQOL-BREF consists of 26 items. The first two items evaluate self-perceived quality of life and the second item on satisfaction with health. Participants’ perception of quality of life is scaled in a positive direction, which means the higher the scores, the better is perceived satisfaction to quality of life. According to Salim, Sudharma, Kusumaratna, and Hidayat (2016), to evaluate perceived quality of life and satisfaction with health, the best cut-off point is 60. However, in this study, a mean score of 50 was used as a cut-off.

The other 24 items were divided into four domains (physical health, psychological aspects, social relationships, and environment). All of the items were scored on a 5-point Likert scale: “very poor to very good” (evaluation scale), “very dissatisfied to very satisfied” (evaluation scale), “none to extremely” (intensity scale), “none to complete” (capacity scale), and “never to always” (frequency scale). The mean score of all items is used to calculate the domain score. Mean scores are then multiplied by 4 in order to make domain scores comparable with the scores used in the WHOQOL-100. Raw scores were first converted to range between 4 and 20 and domain scores were converted to a 0–100 scale with reference to the WHOQOL-BREF Instructions (WHO, 1996). WHOQOL scores of each domain were then divided into two dichotomous categories with mean scores of each domain as a cut-off as follows: 50 for physical health, 51 for psychological, 50 for social relationship, and 58 for environment.

## Results

Table 1 displays the demographic data of the respondents in this study.

**Table 1:** Demographic Characteristics of the Respondents (N = 397)

Characteristics	Frequency	Percentage (%)
Gender		
Male	220	55.4
Female	177	44.6
Education		
Primary	74	18.6
Junior high	91	22.9
Senior high	115	29.0
Higher education	117	29.5
Marital status		
Married	288	72.5
Unmarried	3	0.8
Widower	41	10.3
Widow	65	16.4
Live with		
Spouse	285	71.8
Spouse and/or children	108	27.2
Live alone	4	1.0

The majority of the participants were male (55.4%), married (72.5%), living with a spouse (71.8%) and predominantly having higher education (29.5%).

The participants’ perceptions of health and quality of life were measured based on responses to the first and second questions on the WHOQOL-BREF (Table 2).

**Table 2:** Participants’ Perceptions on their Quality of Life and Health (N = 397)

	Age ≥ 60–74 years	
Variable	Frequency	%
Quality of life		
Poor	22	5.5
Good	375	94.5
Satisfaction to health		
Satisfied	40	10.0
Dissatisfied	357	90.0

Using a score of 50 as the cut-off for question 1 and 2 on the WHOQOL-BREF, the majority of the participants perceived their quality of life to be good (94.5%) and only 5.5% participants perceived it to be poor.

The quality of life was determined based on participants’ responses on 24 items which cover four domains on the WHOQOL-BREF (Table 3).

**Table 3:** Quality of Life of Older People in Four Domains (N = 397)

Domain	Frequency	Percentage (%)
Physical health		
Good	180	45.3
Poor	217	54.7
Psychological		
Stable	219	55.2
Unstable	178	44.8
Social relationships		
Active	156	39.3
Not active	241	60.7
Environment		
Adequate	59	14.9
Inadequate	338	85.1

## Discussion

Quality of life is the extent to which an individual can feel and enjoy all the important events in life in a prosperous way and this concept is often used to measure the well-being of individuals, communities, and nations (Rapley, 2003). This descriptive study involved 397 older participants with a history of essential hypertension, coronary heart disease, heart failure, and ischemic heart failure who were assessed on their quality of life consisting of physical health, psychological aspects, social relationships, and environment. The study results showed that most of the elderly patients in the outpatient department of the hospital where this study was performed demonstrated a good quality of life (94.5%). These results were surprising as it is known that people with heart conditions had impaired quality of life as compared to with healthy population. A study assessing quality of life of 205 patients with congestive heart failure and systolic dysfunction using the generic quality of life measure (SF-36) showed significant lower quality of life in patients with lower cardiopulmonary function as assessed by the New York Heart Association (Juenger et al., 2002).

Individuals with certain heart conditions may experience symptoms that prevent them from maintaining a good quality of life. For example, in a study by Nordgren and Sorensen (2003), patients with chronic heart failure were reported to experience symptoms such as dyspnea, edema, sleeping difficulties, depression, and chest pain with breathlessness as the most common symptom. These physical and emotional symptoms may limit the patients’ daily physical and social activities resulting in poor quality of life (Vaccarino, Kasl, Abramson, & Krumholz, 2001). Moreover, findings from this study were not supported by data reporting that people aged ≥64 years were likely to have a poorer quality of life than those aged <64 years (Pradono et al., 2009). According to Pradono et al. (2009), other demographic factors that affect the quality of life of people aged ≥64 years consist of mental disorders, environmental exposures, and gender. Furthermore, they noted that women were 1.3 times more likely to have a poorer quality of life than men. This present study, however, failed to investigate which gender had a better quality of life.

Having a history of heart disease may affect quality of life of individuals or even lower their welfare. In a previous cohort study in Italy that assessed heart-related quality of life, the participants reported a poor quality of life. The study involved 210 older people who lived in the community and revealed a low quality of life of the participants, who were referred to a geriatric clinic in the city of Milan, as measured by a heart-related quality of life (HRQOL) questionnaire (Bilotta et al., 2011). This study used a pre- and post-test design. At post-test, the study showed that the quality of life of the participants was poor, which was then used by the author as a predictor of nursing home placement and death of the participants within a year (Bilotta et al., 2011).

## Conclusions

This study was conducted with 397 respondents to identify the quality of life of the elderly in the four domains (physical, psychological, environmental, and social relationships) in the outpatient department of a hospital in Banten Province using the WHOQOL-BREF questionnaire. The results showed that most participants with history of CVD (94.5%) had a good quality of life based on environmental aspects, social relationships, physical health, and psychological condition. The measurement tool used in this study assessed quality of life in general. Future research should consider using a quality of life measurement tool with a specific focus on people with CVD diseases, for example, HRQOL, to measure the quality of life in people more accurately.

## Declaration of Conflicting Interests

The authors declared no potential conflicts of interest with respect to the research, authorship, and/or publication of this article.
